# Acute Kidney Injury in Hospitalized Patients Infected with COVID-19 from Wuhan, China: A Retrospective Study

**DOI:** 10.1155/2021/6655185

**Published:** 2021-01-11

**Authors:** Yujie Dai, Zhifen Liu, Xingguo Du, Honglan Wei, Yang Wu, Hua Li, Ming Tian, Chengxu Li, Xiaohong Song, Weicong Wu, Yuan Cai, Yikai Yu, Shaoxian Hu, Junwu Dong

**Affiliations:** ^1^Department of Nephrology and Rheumatology, Puai Hospital, Tongji Medical College, Huazhong University of Science and Technology, Wuhan, Hubei, China; ^2^Department of Rheumatology and Immunology, Tongji Hospital, Tongji Medical College, Huazhong University of Science and Technology, Wuhan, China

## Abstract

**Background:**

Since the first diagnosed case of infection with the novel coronavirus (SARS-CoV-2), there has been a rapid spread of the disease with an increasing number of cases confirmed every day, as well as a rising death toll. An association has been reported between acute kidney injury (AKI) and mortality in patients infected with SARS-CoV-2. Therefore, our study was conducted to explore possible risk factors of AKI as well as whether AKI was a risk factor for worse outcome, especially mortality among patients with coronavirus disease (COVID-19).

**Methods:**

We included all hospital admissions with confirmed or clinically diagnosed COVID-19 from January 29 to February 25, 2020. We collected demographic and epidemiological information, past medical history, symptoms, laboratory tests, treatments, and outcome data from electronic medical records. A total of 492 patients with diagnosed or clinically diagnosed COVID-19 were included in this study.

**Results:**

The prevalence rate of AKI was 7.32%. Among the factors associated with AKI, males versus females (aOR 2.73), chronic kidney disease (aOR 42.2), hypertension (aOR 2.82), increased leucocytes (aOR 6.08), and diuretic use (aOR 7.89) were identified as independent risk factors for AKI among patients infected by SARS-CoV-2. There was a significant difference in hospital fees and death in patients with and without AKI (*p* < 0.05). The mortality rate in patients with AKI was 63.9%.

**Conclusions:**

AKI was widespread among patients with COVID-19. The risk factors of AKI in COVID-19 patients included sex, chronic kidney disease, hypertension, infection, and diuretic use. AKI may be associated with a worse outcome, especially mortality in COVID-19 patients.

## 1. Introduction

On Dec 8, 2019, several cases of pneumonia of unknown cause, now known as severe acute respiratory syndrome, coronavirus-2 (SARS-CoV-2) infection, or coronavirus disease (COVID-19), were reported in Wuhan, Hubei Province, China [1, 2]. Currently, COVID-19 has spread so rapidly that, by 21 April 2020, 2,402,250 confirmed cases and 163,097 deaths were reported by the World Health Organization (WHO). Clinical manifestations of patients infected by SARS-CoV-2 include fever, cough, dyspnea, diarrhea, fatigue and myalgia, normal or decreased leukocyte counts, decreased lymphocyte counts, and radiographic evidence of pneumonia [3, 4]. Although most cases have mild symptoms and a good prognosis, some patients rapidly develop acute respiratory distress syndrome (ARDS), acute respiratory failure, acute cardiac injury, acute kidney injury (AKI), and death [1].

As is known, AKI causes fluid-electrolyte imbalance, fluid overload and impaired metabolic function, neutrophil dysfunction, and immunological dysfunction, which may result in substantial hospitalization expenses and mortality [5, 6]. SARS-CoV-2 has previously been reported to cause kidney dysfunction [7, 8]. However, the risk factors giving rise to AKI in patients infected with SARS-CoV-2 have not been reported to date. Thus, the aim of this study was to determine the risk factors associated with AKI in admission with confirmed or clinically diagnosed with COVID-19. We analyzed the prevalence, risk factors, and outcome of AKI in patients with COVID-19. We believe that the early and rapid diagnosis of AKI might be beneficial for clinicians to assist with the management of patients with COVID-19.

## 2. Methods

### 2.1. Study Site and Participants

This was a hospital-based retrospective, single-center study conducted at Puai Hospital, Tongji Medical College, Huazhong University of Science and Technology, Wuhan, China, which is a designated hospital for SARS-CoV-2 pneumonia. Because the SARS-CoV-2 antibody test was not available in our hospital at the time of the study, the diagnosis of SARS-CoV-2 pneumonia was confirmed by SARS-CoV-2 nucleic acid test using real-time polymerase chain reaction (RT-PCR) based on WHO interim guidance [9]. Clinically diagnosed cases were those who had a clear exposure history to COVID-19, clinical characteristics, chest imaging changes, and in whom common bacterial and viral pathogens that cause pneumonia had been excluded, but without a positive result on SARS-CoV-2 nucleic acid tests during hospitalization. These patients were admitted centrally to the hospital. Four hundred and ninety-two confirmed or clinical diagnoses of SARS-CoV-2 pneumonia inpatients were retrospectively recruited to the study from January 29 to February 25, 2020. The study was approved by Puai Hospital Ethics Committee, and informed consent was waived by the Ethics Commission.

### 2.2. Procedures

**Figure 1:**
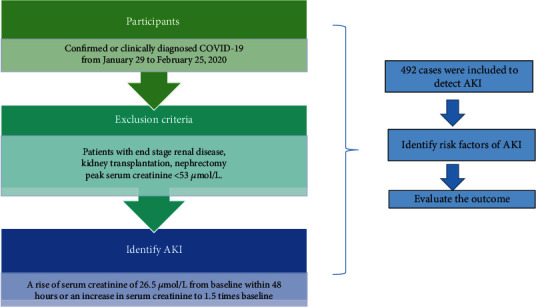
Study profile. We described the study process in the picture. The included participants in this study were those who were confirmed or clinically diagnosed with COVID-19 from January 29 to February 25, 2020. Patients with end-stage renal disease, kidney transplantation, nephrectomy, or peak serum creatinine < 53 *μ*mol/L were excluded. The diagnosis of AKI in this study was a rise of serum creatinine of 26.5 *μ*mol/L from baseline within 48 hours or an increase in serum creatinine to 1.5 times baseline. In the end, we included 492 cases in this study. Next, we identified risk factors of AKI in patients with COVID-19 during hospital admission and then evaluated their outcome. COVID-19 = 2019 coronavirus disease; AKI = acute kidney injury.

All confirmed or clinical diagnoses of COVID-19 hospital admissions were eligible for enrolment into this study (Figure 1). Patients with end-stage renal disease, kidney transplantation, nephrectomy, or peak serum creatinine < 53 *μ*mol/L were excluded. The diagnosis of AKI in this study was established by Kidney Disease Improving Global Outcomes (KDIGO) criteria of a rise of serum creatinine of 26.5 *μ*mol/L from baseline within 48 hours or an increase in serum creatinine to 1.5 times baseline [10]. The rise of serum creatinine is known or presumed to have occurred within the prior seven days. Demographic and epidemiological information including gender, age, exposure history, history of cigarette smoking, and alcohol use was obtained from medical records or from communication with patients. Similarly, past medical history, including a history of chronic kidney disease, hypertension, diabetes, and malignant tumors, was obtained from a review of the medical records. Details of clinical symptoms (including fever, cough, dyspnea, and diarrhea) and laboratory tests (including a complete blood count, serum creatinine, C-reactive protein (CRP), procalcitonin (PCT), and nasopharyngeal swabs tested for SARS-CoV-2 using RT-PCR assays) were available from electronic medical records. Details of treatments including antibiotics, antiviral agents, hormones, and nonsteroidal anti-inflammatory drugs could be obtained from medical records. The clinical outcomes of the included cases were ended up to February 25, 2020. Besides the obvious improvement of both clinical symptoms and pulmonary inflammation on chest computed tomography (CT), it was required that at least two consecutive SARS-CoV-2 nucleic acid tests were negative before hospital discharge.

### 2.3. Data Collection

All data were collected and checked by two doctors (YD and ZL), independently. Some data that are missing or unavailable from electronic medical records were acquired by directly communicating with attending doctors or patients or their families.

### 2.4. Statistical Analysis

We used SPSS (version 17.0) to enter and analyze data. Continuous variables were expressed as mean (standard deviation (SD)) if they fit the normal distribution and were compared with Student's *t*-test or variance analysis. Median (interquartile range (IQR)) was used if they were not normally distributed and were compared with the Mann–Whitney *U* test. Categorical variables were presented as count (%) and compared with the chi-square or Mann–Whitney *U* test. Binary logistic regression was performed to determine the association between the different associated factors and AKI. Orderly logistic regression was then used to examine the association between multiple variables and different stages of AKI. A *p* value of <0.05 was considered statistically significant.

### 2.5. Role of the Funding Source

The funders of this study played no role in the study design, data collection, data analysis, data interpretation, or writing of the report. The corresponding author had full access to all the data in the study and had final responsibility for the decision to submit for publication.

## 3. Results

### 3.1. Prevalence of AKI and Baseline Characteristics of Patients with COVID-19

**Figure 2:**
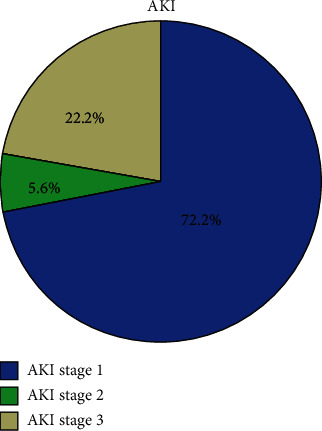
The proportion of different stages of AKI. AKI was staged according to severity based on the KDIGO guideline. Of these patients, 8 (22.2%), 2 (5.6%), and 26 (72.2%) were diagnosed with stages 3, 2, and 1 kidney disease, respectively. AKI = acute kidney injury; KDIGO = Kidney Disease Improving Global Outcomes.

A total of 492 patients with COVID-19 that were admitted to our hospital were included in this study. 249 patients had positive SARS-CoV-2 nucleic acid test, and 243 patients had a negative result. Among all patients, 36 (7.32%) patients were diagnosed with AKI. Of these patients, 8 (22.2%), 2 (5.6%), and 26 (72.2%) were diagnosed with stages 3, 2, and 1 kidney disease, respectively (Figure 2). Of the 36 COVID-19 patients with AKI, 24 (66.67%) were men and 12 (33.33%) women (Table 1). Most patients were elderly with a mean age of 69 (59-78) years. There are 5 (13.89%) patients with a smoking history and 4 (11.1%) with a history of alcohol use. Most patients had chronic diseases, including chronic kidney disease 4 (11.1%), hypertension 21 (58.33%), diabetes 9 (25%), and malignant tumors 3 (8.33%). The common symptoms at onset of illness were fever 27 (75%), cough 27 (75%), and shortness of breath 19 (52.78%); a less common symptom was diarrhea 2 (5.56%).

### 3.2. Laboratory Tests of Patients with and without AKI

When patients were detected as having AKI, the median white blood cell count was 9.32 × 109 per L (IQR 4.85-16.31); 17 (47.22%) patients were above the normal range and 5 (13.89%) below. Compared to patients without AKI, more patients with AKI had increased leucocyte level (*p* < 0.001) (Table 2). The median lymphocyte count was 0.59 × 10^9^/L (IQR 0.36-1.23) in 36 patients; levels of lymphocyte count were above the normal range in one (2.78%) and below the normal range in 27 (75%). The percentage of lymphocyte count in patients with AKI was higher than that in those without (*p* < 0.001). The median neutrophil count was 7.64 × 10^9^/L (IQR 3.61-15.24) in patients confirmed with AKI; 18 (50%) had levels above the normal range and 3 (8.33%) below. Similarly, patients with AKI had a higher rate of neutrophils compared to patients without AKI (*p* < 0.001). The median PCT was 0.29 (IQR 0.07-1.66) and CRP 45.97 (15.98-107.73) in patients diagnosed with AKI. In the SARS-CoV-2-infected patients, those with AKI had a significantly higher PCT and CRP than those without (*p* < 0.001). This suggests secondary bacterial infection in those patients with AKI.

### 3.3. Treatments of Patients with and without AKI

All patients were treated in isolation. In those patients diagnosed with AKI, 11 (30.56%) received diuretic treatment for heart failure or edema (Table 3). More patients in the AKI group received diuretic treatment than those without AKI (*p* < 0.001). Most patients received empirical antibiotic and antiviral treatment after admission; 34 (94.44%) patients received quinolone therapy and 8 (22.22%) received penicillin therapy. Thirty-four (94.44%) patients received antiviral therapy, including oseltamivir, ribavirin, ganciclovir, and arbidol. Significantly more patients (63.89%) with AKI had received systematic corticosteroids at some point compared to those without AKI (*p* < 0.001). Eight (22.22%) patients had taken nonsteroidal anti-inflammatory drugs (NSAIDs) for fever reduction. Twenty-three (63.89%) patients received proton pump inhibitors (PPIs); there was a statistically significant difference in the number of patients with AKI who had received PPIs compared to those without AKI (*p* < 0.05). Two (5.56%) patients received traditional Chinese medicine.

### 3.4. Risk Factors for AKI in Patients with COVID-19

Sex, age, history of smoking and alcohol, and past medical history were included in the logistic regression analysis (Table 4). Considering that clinical symptoms such as fever and diarrhea may be related to AKI, both were evaluated in the logistic regression analysis. Besides these factors, treatment and infection indicators, including blood count, CRP, and PCT, were also analyzed. Logistic regression modeling demonstrated that males versus females (adjusted odds ratio (aOR) 2.73, 95% confidence intervals (CI) 1.05-7.13), chronic kidney disease (aOR 42.2, 95% CI 3.94-452.37), hypertension (aOR 2.82, 95% CI 1.08-7.34), increased leucocytes (aOR 6.08, 95% CI 1.58-23.35), diuretic use (aOR 7.89, 95% CI 2.51-24.78), and glucocorticoids (aOR 4.36, 95% CI 1.71-11.16) were independent risk factors for the development of AKI among confirmed or clinically diagnosed COVID-19 inpatients. To further analyze the risk factors for the severity of AKI, we included all these factors into an orderly logistic regression model. However, none of them had statistical significance.

### 3.5. Outcomes of Patients with and without AKI

**Figure 3:**
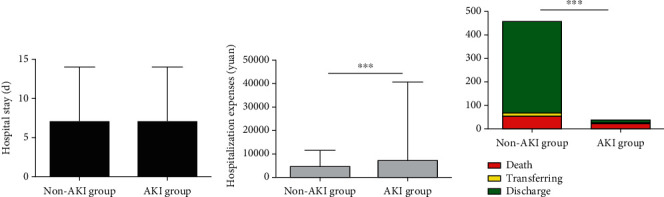
The outcome of the non-AKI group and the AKI group. Patients with AKI had a slightly longer hospital stay than those without, but it has no statistical significance (a). Participants with AKI had significantly higher hospitalization fees than those without and with a statistical significance (*p* < 0.001) (b). Patients with AKI had a much higher mortality rate than those without AKI (63.9% versus 11.4%; *p* < 0.001) (c). ^∗^*p* < 0.05, ^∗∗^*p* < 0.01, and ^∗∗∗^*p* < 0.001. AKI = acute kidney injury.

Finally, the hospital stay, hospitalization expenses, and outcome were evaluated using nonparametric tests (Table 5). Patients with AKI had a slightly longer hospital stay than those without: 11 [7–14] versus 10 days [7–14], which was not statistically significant. Participants with AKI had significantly higher hospitalization fees than those without: 7353.37 yuan (4657.50-11578.42) versus 15500.73 yuan (7170.23-40778.82) (*p* < 0.001). There was a much higher mortality rate in patients with AKI than those without AKI (63.9% versus 11.4%; *p* < 0.001). The mortality rates of patients with AKI stages 1, 2, and 3 were 53.84%, 50%, and 100%, respectively. This suggests that AKI is associated with in-hospital death. Eleven of the 36 (30.56%) patients with AKI had recovered and were discharged, while 390 (85.5%) patients in the non-AKI group had been discharged or discontinued isolation (Figure 3).

## 4. Discussion

Previously published research reported that SARS-CoV-2 might cause damage to organs other than the lungs, such as AKI and cardiovascular injury [1]. AKI, a common disorder with a high risk of development of chronic kidney disease and mortality, is easily overlooked by doctors [6]. A multicenter study reported that 7% of all hospital admissions were suspected of having AKI after screening 2,223,230 patients from 44 hospitals in China in 2013 [11]. Their survey also suggested that the detection rate of AKI was 0.99% according to the KDIGO criteria and 2.03% according to expanded criteria. Some single-center studies have also reported an estimated prevalence of AKI in hospitalized patients between 2.41% and 3.19% [12, 13], which is much lower than in developed countries (7–18%) [14–17]. Here, we detected a rate of 7.32% among clinically diagnosed or confirmed SARS-CoV-2 pneumonia inpatients, which is significantly higher than the previously reported prevalence in China. Moreover, it has previously been reported that the rate of AKI in patients with COVID-19 is between 3% and 9% [1, 7], suggesting that these patients have a high risk of developing AKI.

In view of the prevalence of AKI among clinically diagnosed or confirmed COVID-19 inpatients, the factors contributing to its development need to be explored. Based on the above discussion, we assessed that patients who were male, >60 years old, had a history of smoking and alcohol use, or had a past medical history of chronic kidney disease, hypertension, or diabetes had a significantly higher risk of acquiring AKI (*p* < 0.05). Other factors such as laboratory tests (increased leucocytes, decreased lymphocytes, increased neutrophils, PCT, and CRP) and drugs (diuretics, glucocorticoids, and PPIs) also significantly correlated with the detection of AKI (*p* < 0.05). We used a binary logistic regression to determine the association between the different associated factors and AKI. In this model, men, chronic kidney disease, hypertension, secondary bacterial infection, diuretic, and glucocorticoid use were identified as independent risk factors for the development of AKI among all confirmed or clinically diagnosed COVID-19 cases treated in our hospital. Little information is available regarding nephrotoxicity caused by glucocorticoids. After screening the electronic medical records of all hospital admissions, we found that glucocorticoid treatment was more frequently used in critically ill patients, possibly for acute respiratory distress syndrome, shock, or decreased immune response. Those conditions also accounted for the high rate of AKI and possibly explain the higher use of glucocorticoid use in the AKI group.

There is no evidence to prove kidney injury is directly due to SARS-CoV-2 itself; however, some studies indirectly support kidney damage in SARS-CoV-2 infection. First, the detection of PCR fragments of SARS-CoV-2 in the blood of infected patients may play a pathogenic role in kidney damage [1]. It has recently been reported that angiotensin-converting enzyme 2 (ACE2) is used as a cell entry receptor by SARS-CoV-2, similar to the case with SARS-CoV [18–20]. The expression of ACE2 in the kidney is nearly 100 times higher than in the lung [8]. Therefore, SARS-CoV-2 may cause kidney damage via the ACE2 pathway. However, currently there is not a large set of kidney biopsy specimens to confirm this hypothesis. Further study is required as to whether the coronavirus exerts effects directly on renal tissue.

It has been reported that AKI is correlated with negative clinical outcomes, such as death, prolonged hospital stay, and increased hospital costs [15]. In our study, there was no difference in the length of hospital stay between clinically diagnosed or confirmed COVID-19 patients with and without AKI. This is probably related to the discharge criteria. Apart from the obvious improvement in both clinical symptoms and pulmonary inflammation, all patients were required to stay in the hospital until at least two consecutive SARS-CoV-2 nucleic acid tests were negative. As patients with AKI had a longer period of recovery, there was a significant difference in hospital fees and death between those with and without AKI (*p* < 0.001). The mortality rate in clinically diagnosed or confirmed COVID-19 patients with AKI was 63.9%, while in those without AKI it was 11.4%. AKI has become a significant risk factor for mortality among COVID-19 admissions. Therefore, it is important for doctors to be alert to the development of AKI when treating COVID-19 patients.

Previous studies reported the prevalence of AKI in patients infected with SARS-CoV-2 and the fact that this was associated with a high mortality rate [1, 7]. We first analyzed the risk factors associated with the development of AKI during hospitalization in patients with SARS-CoV-2. We then discussed the clinical characteristics and outcomes of AKI in patients with confirmed or clinically diagnosed COVID-19 compared to those without AKI.

This study has several limitations. First, the serum creatinine measurements were not performed frequently enough so that a diagnosis of AKI could not be confirmed in some patients. For example, a rise of serum creatinine of 26.5 *μ*mol/L from baseline occurred in a period of more than 48 hours. This could have resulted in a lower detection rate of AKI in the admissions. Second, an accurate baseline serum creatinine level was not available in most patients, as the majority did not check their creatinine levels regularly. This might have resulted in an underestimate of the prevalence of chronic kidney disease or decrease in the prevalence of AKI. Therefore, further research is required to confirm our results.

In conclusion, the prevalence of AKI was higher in patients confirmed or clinically diagnosed with COVID-19 compared to conditions not related to the COVID-19 pandemic in China. Male sex, chronic kidney disease, hypertension, secondary bacterial infection, and diuretic use were identified as independent risk factors for AKI in confirmed or clinically diagnosed COVID-19 infected hospital admissions. The occurrence of AKI is a poor prognostic factor in SARS-CoV-2-infected patients. The mortality rate in COVID-19 patients complicated with AKI group is very high. Therefore, early identification of risk factors and timely treatment of AKI in SARS-CoV-2-infected patients are important. Our study provides reliable data on the prevalence of AKI, depicts its characteristics, and shows how to recognize the risk factors of AKI in SARS-CoV-2-infected patients. This may help clinicians to reduce the prevalence of AKI and thereby decrease the mortality rate in patients with SARS-CoV-2 infection.

## Figures and Tables

**Table 1:** Demographics and baseline characteristics of patients infected with SARS-CoV-2 (*N* = 492).

	Number (%)	Patients with AKI (*n* = 36)	Patients without AKI (*n* = 456)	*p* value
Gender				
Male	226 (45.93%)	24 (66.67%)	202 (44.30%)	0.01
Female	266 (54.07%)	12 (33.33%)	254 (55.70%)	

Age (y)				
≤35	44 (8.94%)	1 (2.78%)	43 (9.42%)	<0.001
35–60	215 (43.70%)	8 (22.22%)	207 (45.39%)	
≥60	233 (47.36%)	27 (75.00%)	206 (45.18%)	

Smoking	29 (5.89%)	5 (13.89%)	24 (5.26%)	0.03
Alcohol	17 (3.46%)	4 (11.11%)	13 (2.85%)	0.01
Chronic kidney disease	6 (1.22%)	4 (11.11%)	2 (0.44%)	<0.001
Hypertension	141 (28.66%)	21 (58.33%)	120 (26.32%)	<0.001
Diabetes	65 (13.21%)	9 (25.00%)	56 (12.28%)	0.03
Malignant tumor	19 (3.86%)	3 (8.33%)	16 (3.51%)	0.15
Fever	383 (77.85%)	27 (75.00%)	356 (78.07%)	0.67
Cough	339 (68.90%)	27 (75.00%)	312 (68.42%)	0.41
Shortness of breath	174 (35.37%)	19 (52.78%)	155 (33.99%)	0.02
Diarrhea	54 (10.98%)	2 (5.56%)	52 (11.40%)	0.28

Data are median (IQR), or *n*/*N* (%), where *N* is the total number of patients with available data. *p* values comparing patients with AKI and without AKI are from chi-square or Mann–Whitney *U* test. SARS-CoV-2 = 2019 novel coronavirus; AKI = acute kidney injury.

**Table 2:** Laboratory tests of patients infected with SARS-CoV-2 (*N* = 492).

Laboratory tests		Patients with AKI	Patients without AKI	*p* value
Leucocytes (×109 per L; normal range 3.5–9.5)	Mean	9.32 (4.85-16.31)	5.03 (3.93-6.28)	<0.001
	Normal	14 (38.89%)	352 (77.19%)	
	Increased	17 (47.22%)	27 (5.92%)	
	Decreased	5 (13.89%)	77 (16.89%)	

Lymphocytes (×109 per L; normal range 1.1–3.2)	Mean	0.59 (0.36-1.23)	1.11 (0.8-1.48)	<0.001
	Normal	8 (22.22%)	225 (49.34%)	
	Increased	1 (2.78%)	5 (1.10%)	
	Decreased	27 (75%)	226 (49.56%)	

Lymphocyte percentage (20-50%)	Mean	6.6 (3.38-23.8)	24.15 (16.7-32.4)	<0.001
	Normal	9 (25%)	289 (63.40%)	
	Increased	2 (5.56%)	4 (0.88%)	
	Decreased	25 (69.44%)	163 (35.75%)	

Neutrophils (×109 per L; normal range 1.8–6.3)	Mean	7.64 (3.61-15.24)	3.2 (2.31-4.47)	<0.001
	Normal	15 (41.67%)	354 (77.63%)	
	Increased	18 (50%)	47 (10.31%)	
	Decreased	3 (8.33%)	55 (12.06%)	

Neutrophil percentage (40-75%)	Mean	87.95 (67.83-92.55)	65.6 (56.93-74.28)	<0.001
	Normal	10 (27.78%)	337 (73.90%)	
	Increased	24 (66.67%)	111 (24.34%)	
	Decreased	2 (5.56%)	8 (1.75%)	

PCT (ng/L)	Mean	0.29 (0.07-1.66)	0.04 (0.02-0.07)	<0.001
	<0.5	26 (72.22%)	440 (96.49%)	
	0.5-2	5 (13.89%)	11 (2.41%)	
	2-10	5 (13.89%)	4 (0.88%)	
	≥10	0.00	1 (0.22%)	

CRP (mg/L)	Mean	45.97 (15.98-107.73)	15.9 (3.61-49.23)	<0.001
	0-10	8 (22.22%)	182 (39.91%)	
	10-25	5 (13.89%)	85 (18.64%)	
	25-50	7 (19.44%)	77 (16.89%)	
	50-100	6 (16.67%)	78 (17.11%)	
	≥100	10 (27.78%)	34 (7.46%)	

Data are median (IQR), or *n*/*N* (%), where *N* is the total number of patients with available data. *p* values comparing patients with AKI and without AKI are from chi-square or Mann–Whitney *U* test. SARS-CoV-2 = 2019 novel coronavirus; AKI = acute kidney injury; PCT = procalcitonin; CRP = C-reactive protein.

**Table 3:** Treatment of patients infected with SARS-CoV-2 (*N* = 492).

Treatment	Patients with AKI	Patients without AKI	*p* value
Diuretic	11 (30.56%)	18 (3.95%)	<0.001
Quinolone	34 (94.44%)	389 (85.31%)	0.13
First- or second-generation cephalosporins	0.00	22 (4.82%)	0.18
Penicillin	8 (22.22%)	88 (19.30%)	0.67
Antiviral	34 (94.44%)	393 (86.18%)	0.16
Glucocorticoid	23 (63.89%)	135 (29.61%)	<0.001
NSAID	8 (22.22%)	108 (23.68%)	0.84
PPI	23 (63.89%)	212 (46.49%)	0.04
Traditional Chinese medicine	2 (5.56%)	43 (9.43%)	0.44

Data are *n*/*N* (%), where *N* is the total number of patients with available data. *p* values comparing patients with AKI and without AKI are from chi-square or Mann–Whitney *U* test. SARS-CoV-2 = 2019 novel coronavirus; AKI = acute kidney injury; NSAID = nonsteroidal anti-inflammatory drugs; PPI = proton pump inhibitor.

**Table 4:** Binary logic regression on risk factors for AKI (*N* = 492).

Binary logic regression on risk factors for AKI (*n* = 492)				
Risk factor	Standard error	Wald *χ*^2^	95% CI	*p* value
Gender (M/F)	0.49	4.22	2.73 (1.05-7.13)	0.04
Chronic kidney disease	1.21	9.56	42.2 (3.94-452.37)	0.002
Hypertension	0.49	4.49	2.82 (1.08-7.34)	0.034
Leucocytes (increased/normal)	0.69	6.91	6.08 (1.58-23.35)	0.009
Diuretic	0.58	12.53	7.89 (2.51-24.78)	<0.001
Glucocorticoid	0.48	9.44	4.36 (1.71-11.16)	0.002

Data are median (IQR). *p* values comparing patients with AKI and without AKI are from binary logic regression with backward selection. SARS-CoV-2 = 2019 novel coronavirus; AKI = acute kidney injury.

**Table 5:** The outcome of patients infected with SARS-CoV-2 (*N* = 492).

	Patients with AKI	Patients without AKI	*p* value
Hospital stay (d)	11 (7-14)	10 (7-14)	0.46
Hospitalization expenses (yuan)	15500.73 (7170.23-40778.82)	7353.37 (4657.50-11578.42)	<0.001

Clinical outcome			
Death	23 (63.9%)	52 (11.4%)	<0.001
Discharge	11 (30.56%)	390 (85.5%)	
Transferring	2 (5.6%)	14 (3.1%)	

Data are median (IQR), or *n*/*N* (%), where *N* is the total number of patients with available data. *p* values comparing patients with AKI and without AKI are from chi-square or Mann–Whitney *U* test. SARS-CoV-2 = 2019 novel coronavirus; AKI = acute kidney injury.

## Data Availability

We would put our data on public website later.
